# Case of Doubtful Paternity

**Published:** 1852-10

**Authors:** W. L. Sutton

**Affiliations:** President of the Medical Society of Kentucky; Georgetown


### Art. ii.—*A Case of Doubtful Paternity.* By W. L. Sutton, M. D., President of the Medical Society of Kentucky.

*What distinguishes a child of pure white blood from one tainted with that of the negro?*

About a fortnight ago a child was brought to Georgetown by its reputed father, accompanied by his physician, a gentleman of some forty-five or fifty years, for examination by the physicians of the town, partly with a view of ascertaining whether any course could be suggested which would insure the continuance of its life, and partly to silence some neighborhood talk which had arisen on account of its color. The physician believed that the color of the child was occasioned by the foramen ovale remaining open; in proof of this, he alleged that when the child cried he became much darker—decidedly blue—and thought that the imperfect aëration of the blood consequent upon the patent condition of the foramen, was sufficient to account for the permanent dark color of the skin. Another physician who had seen the child at two months old, believed then that he labored under inflammation of the brain, and, from some cause, suspected the foramen ovale was open. Among other things he suggested a trial of the advice of Prof. Meigs, respecting position.

The moral testimony in the case was, that up to the birth of the child, the mother had been entirely above suspicion. In fact, she was considered a very modest woman. She was said to have fair complexion, light hair, and blue eyes. The husband, who accompanied the child, had nothing remarkable as to complexion; hair of the ordinary brownish color. His mother reported to be very dark, with black hair.

The child is a boy of four months, with black, straight hair, the fine hair on the forehead black; rounded forehead, broad nose, particularly expanded at the alæ, skin dark, yet not darker than purely white children are sometimes seen. Near the extremity of the coccyx, and rather to one side, was a spot, oval in shape, about three-fourths of an inch long, decidedly dark. No other dark spot perceived. There is a popular notion that when a child is tainted with African blood, the scrotum and a streak down the back are always dark. Nothing of that kind existed.

Three physicians (the one who had seen the child at two months, among them,) were unanimous that the color did not depend on cyanosis, and that there were appearances about the child of very suspicious tendency; but declined any expression as to admixture of blood, without a better acquaintance with the relatives of the husband and wife. One, however, could not believe that two parents of the temperament of the husband and wife, could produce a child of that color. Another thought it possible that with a grandmother decidedly dark, a child might be even that dark; but could see no good reason why a child purely white should have such a nose. The third thought that although the appearances were suspicious, they were not enough so to give an opinion unfavorable to a woman as free from suspicion as the mother had been.

The family physician, who had unshaken faith in the chastity of the woman, expressed his unfeigned astonishment that any hesitation should be felt in giving an opinion tending to exculpate the mother from all suspicion. Not satisfied with the result of this consultation, he procured the attendance of some five or six others at the residence of the parties. These last, I understand, took very much the same view of the case that had been taken in the consultation.

Subsequently it is reported that the woman acknowledged that she had had occasional connection with two negro men in the neighborhood.

As this is a point which has been but little investigated, (some little discussion in Beck's Medical Jurisprudence being all that I am aware of,) it may not be amiss to make a few comments. Many persons think nothing would be easier than to "tell a white child from a black one;" but like a great many other things, the more it is studied, the more difficulties start up. From the nature of things, our European brethren give us no authority upon this point. Beck, vol. 1, p. 485, gives us a case which occurred in New York. In this case a mulatto woman swore a child to a black man. On trial, when the child was one year and seven months old, it appeared in evidence "that the child was somewhat dark, but lighter than the generality of mulattoes; and that its hair was straight, and had none of the peculiarities of the negro race. Many of the most eminent members of the medical profession were examined, and they all, with the exception of Dr. Mitchill, declared that its appearance contradicted the idea that it was the child of a black man. Dr. Mitchill, for various reasons, placed great faith in the oath of the female, and persisted in his belief of its paternity, although he allowed its appearance was an anomaly. The Mayor (Hon De-Witt Clinton) and the Court decided in favor of Whistelo," *i. e.* that the child was by a white man.

Dunglison ridicules the opinion of Dr. Mitchill. But Mitchell was altogether as respectable in the profession, and probably as cautious as Dunglison. Beck comments upon the above case as follows: "It will not do, however, to extend this rule too positively with what may be called *mixed breed.*"

Parsons gives an account in the Philosophical Transactions of a black man married to an English woman, of whom the offspring was quite black. In a similar case, the child resembled its mother in fairness of features, and indeed the whole skin was white, except some spots on the thigh, which were as black as the father.

White, in his work on the *Gradation of Man*, mentions a negress who had twins by an Englishman. One was perfectly black, its hair short, woolly, and curled; the other was white, with hair resembling that of an European.

So, also, Dr. Winterbottom knew a family of six persons, one-half of which were almost as light colored as mulattoes, while the other was jet black. The father was a deep black, the mother a mulatto.

"The offspring of a black and white," says Lawrence, "may be either black or white, instead of being mixed, and in some rare cases it has been spotted."

I have made the above extensive extract from Beck, (which is all he says on the subject,) because of the liability of such cases to arise in this community, where we have not only the white and the black races, but every conceivable degree of mixture. In a note to the above extract Beck adverts to the fact that, "At birth it [the new-born black infant] cannot be always distinguished from the white; its hair has not yet its peculiaa make, and we can only notice the tendency to dark on some parts of the body. In a few days, however, the change commences on the countenance, and gradually extends over the body." This is rather too positive. In many cases, a child of purely black parents is so white at birth as to exhibit no "tendency to dark" on any part of the body; and like other changes, this sometimes takes place much more slowly than in others.

There is much truth in the extracts made by Beck above, as careful examination of our mulattoes will shew. In this town there is a family—the father half white, the mother three-fourths—whose children vary very much in color. Some being pretty good samples of the negro, and others, at five or six years old, not only as white as most white children, but having straight and light-colored hair.

The spots spoken of in the above extracts are certainly rare; nor do I know to how much consideration they are entitled. There was one on the child which gives rise to these remarks. On the other hand, without being able at this time to refer to any particular case, I am certainly under the impression that I have seen persons, entirely free from suspicion of admixture, who had a dark spot on some part of the body. I, some time since, owned a negress who clearly had no white blood in her, yet she had a large spot on the forehead and temples greatly darker than her skin in other parts.

The hair, although a very important feature, is not conclusive in determining our judgment. It does not necessarily begin to assume its distinctive character in a few days, as we might infer from the expression of Beck. In half-breeds, generally, it is only curly, and not knappy, as in the negro; frequently it is no more curly than occurs occasionally in persons purely white; whilst again it is as knappy as in the negro.

It seems that no reliance can be placed upon the popular notion that the scrotum and skin over the spine are dark in children having an admixture of African blood. I have examined several children of six to eight years old without finding it in any of them; they were, however, more than half white—some two-thirds, some three-fourths.

We must pay some attention to what is called moral testimony, but that, like other considerations, must be watched. The above case shows how guilty a woman may be, and yet escape suspicion.

In stating facts, I have gone upon the presumption that they were really as they appear. For instance, in the family referred to as living in this town. Some of the children *may* be by fathers purely white, and others by those wholly black. I can only say that no suspicion attaches.

The case cited in which an Englishman impregnated a negress, one child being white and the other black, may be as reported, yet we have a case reported of a woman in Virginia having connection with her husband, and very soon afterwards with a negro man, and becoming impregnated by both.

There is, perhaps, no subject connected with our profession which presents more knotty points than this. For this reason—and because no one knows when he may be consulted upon such a case, and further, as we have so little authority on this point, and because the country South of Mason & Dixon's line could and ought to furnish the facts and authority upon this subject—I have thrown together the above facts and suggestions, in hope that they may be a means of drawing others out on the same subject.

GEORGETOWN, Aug. 18, 1852.

